# Admissions to acute adolescent psychiatric units: a prospective study of clinical severity and outcome

**DOI:** 10.1186/1752-4458-5-1

**Published:** 2011-01-06

**Authors:** Ketil Hanssen-Bauer, Sonja Heyerdahl, Trond Hatling, Gunnar Jensen, Pål Marius Olstad, Tormod Stangeland, Tarje Tinderholt

**Affiliations:** 1 Centre for Child and Adolescent Mental Health, Eastern and Southern Norway, PO Box 4623 Nydalen, N-0405 Oslo, Norway; 2 Department of Research and Development, Division of Mental Health Services, Akershus University Hospital, Lørenskog, Norway; 3 SINTEF Health research, Trondheim, Norway; 4 Adolescent Acute Ward, Nordlandssykehuset, Bodø, Norway; 5 Adolescent Acute Ward, St. Olav University hospital, Trondheim, Norway; 6 Adolescent Psychiatric Clinic, Division of Mental Health Services, Akershus University Hospital, Lørenskog, Norway; 7 Adolescent Psychiatric Acute Unit, Oslo University Hospital, Norway

## Abstract

**Background:**

Several countries have established or are planning acute psychiatric in-patient services that accept around-the-clock emergency admission of adolescents. Our aim was to investigate the characteristics and clinical outcomes of a cohort of patients at four Norwegian units.

**Methods:**

We used a prospective pre-post observational design. Four units implemented a clinician-rated outcome measure, the Health of the Nation Outcome Scales for Children and Adolescents (HoNOSCA), which measures mental health problems and their severity. We collected also data about the diagnoses, suicidal problems, family situations, and the involvement of the Child Protection Service. Predictions of outcome (change in HoNOSCA *total score*) were analysed with a regression model.

**Results:**

The sample comprised 192 adolescents admitted during one year (response rate 87%). Mean age was 15.7 years (range 10-18) and 70% were girls. Fifty-eight per cent had suicidal problems at intake and the mean *intake *HoNOSCA *total score *was 18.5 (SD 6.4). The largest groups of main diagnostic conditions were *affective *(28%) and *externalizing *(26%) *disorders*. Diagnoses and other patient characteristics at intake did not differ between units. Clinical psychiatric disorders and developmental disorders were associated with severity (on HoNOSCA) at intake but not with outcome. Of adolescents ≥ 16 years, 33% were compulsorily admitted. Median length of stay was 8.5 days and 75% of patients stayed less than a month. Compulsory admissions and length of stay varied between units. Mean change (improvement) in the HoNOSCA *total score *was 5.1 (SD 6.2), with considerable variation between units. Mean discharge score was close to the often-reported outpatient level, and *self-injury *and *emotional symptoms *were the most reduced symptoms during the stay. In a regression model, unit, high HoNOSCA *total score *at intake, or involvement of the Child Protection Service predicted improvement during admission.

**Conclusions:**

Acute psychiatric in-patient units for adolescents effectively meet important needs for young people with suicidal risks or other severe mental health problems. These units may act in suicide prevention, stabilizing symptom severity at a lower level within a short stay. It is important to explore the differences in outcome, compulsory admissions, and length of stay between units.

## Background

During the last few decades, the Norwegian Regional Health Authorities have established acute psychiatric in-patient units for adolescents nationwide. These units provide services for the entire adolescent population and are governmentally funded public health services. All these acute wards accept around-the-clock emergency admissions of adolescents with mental health problems or disorders who are in urgent need of hospital care. The treatment is comprehensive and age customized, with a complex set of multiple interventions provided, with the environment of the ward milieu and specialized education replacing the patient's local environment during the admission period. The main goal of these acute units is to stabilize the adolescents by reducing any acutely increased psychiatric symptoms, risk of suicide, and harm to others to a non-problematic level or to a level treatable by outpatient child and adolescent mental health services (CAMHS), by a general practitioner (GP), or by another community-based service. If required, the acute units may refer the adolescents to other in-patient units that provide less-intensive care and longer stays. The services are part of a continuum-of-care model linked to outpatient treatment and community-based services. We do not know of any studies that have clarified the characteristics of adolescents treated in such acute psychiatric in-patient units that accept mainly emergency admissions. In England and Wales, only a few emergency in-patient CAMHS have been organized as separate units [1]. Other in-patient CAMHS units had low capacities for emergency and out-of-hours admissions and turned away the majority of referrals for emergency admission in 2005 [2]. Whether emergency services should be organized as separate acute units is a topic of discussion in the United Kingdom (UK) [3].

There are some observational studies, but few experimentally designed outcome studies of in-patient mental health treatments of children and adolescents [4-11]. A review from 1990 by Pfeiffer and Strzelecki [5] found that psychiatric hospitalization of children and adolescents was often beneficial, particularly if a specialised treatment program and aftercare were available. They found that less pathological clinical picture (organicity, diagnoses and symptom pattern), better family functioning and family involvement in therapy were positive predictors of outcome. IQ and length of stay had a modest positive relationship with outcome. Two more recent studies of in-patient treatment of children and adolescents by Green et al [8,10] also found positive outcome which was predicted by longer stays, positive therapeutic alliance and better premorbid family functioning. Two studies of younger children did not find that involvement of child protection service, length of stay or diagnose predicted outcome [12,13]. These findings indicate that both characteristics of the patient, the family and the treatment program are predictors of outcome, but there are few studies, with varying design and results. In a review of in-patient services, Gowers and Rowlands [14] concluded in 2005 that "although reports show increasing evidence of good clinical outcomes, ... uncertainties remain about the benefits of hospital admission over good quality assertive community-based interventions." Other studies have raised concerns regarding whether admission to a psychiatric hospital results in a dependence on the hospital environment and increased stigma, and whether the benefits and health gains are difficult to transfer to the home situation [15]. We have found no studies that have specifically focused on the outcomes of acute in-patient psychiatric services for adolescents. A recent review of the alternatives to in-patient psychiatric units for children and adolescents concluded that in-patient units are still valuable: "Current evidence suggests that the adolescent in-patient unit has a particular ability to provide stabilization and rapid reduction of symptoms and risks," and that "intensive community treatment is unlikely to fully replace hospitalization" [16]. In-patient treatment is very expensive. In the United States, the population-based discharge rates from in-patient mental health services for children and adolescents did not change between 1990 and 2000, but the total number of in-patient days fell by approximately one half and the median length of stay declined from 12.2 days to 4.5 days, despite higher rates of serious illness and self-harm [17]. A general trend in the UK towards more cost-effective services with shorter in-patient stays has raised the concern that such short stays may influence the therapeutic milieu and reduce the success of outcomes [9,18]. A study in the United States monitored and compared the outcomes of several in-patient services and revealed important differences in service outcomes and quality [19]. The current evidence base provides very little guidance for the development of in-patient CAMHS or alternative services, and it has been suggested that several units be audited to increase the current level of evidence available because randomized controlled trials may be difficult to implement [15]. A recent editorial in the *British Journal of Psychiatry *regarding the accountability of specialist CAMHS underlined the importance of service auditing and the routine measurement of health outcomes [20]. A clinician-rated measure, the Health of the Nation Outcome Scales for Children and Adolescents (HoNOSCA) [21], was developed in the UK for the routine measurement of outcomes in clinical settings. The HoNOSCA has been well evaluated and found to be valid and feasible, to have substantial inter-rater reliability, and to be congruent with parent and referrer outcome ratings [22-28]. It has been used in several in-patient studies [8,29-33]. The HoNOSCA has been widely used in CAMHS to routinely measure outcomes [28,34-38].

The aim of this study was to investigate the patients at four acute in-patient psychiatric units for adolescents in terms of: 1) the characteristics of the patients at admission, 2) their outcomes at discharge and 3) the predictors of outcome. The four units for adolescents investigated in this study also participated in a larger network and multicentre study of acute psychiatric units for adults and adolescents in Norway [39].

## Methods

### Setting

In 2005, there were 16 acute in-patient psychiatric services for adolescents in Norway, with 126 beds. Five of these units established a collaboration to evaluate the services, and they participated in a Norwegian network of acute psychiatric units and teams. One of the adolescent units participating in the network was not included in this study because they mainly worked as an outreach and intensive outpatient service. The four units in the study had a total of 31 beds (range 5-14). All the adolescent in-patient units were responsible for a specific catchment area in which they were the only unit providing psychiatric emergency admissions. In regulations pursuant to the Norwegian Mental Health Act (section 2) [40], the following conditions are specified as qualifying a person for necessary assessment and treatment in an acute psychiatric service without delay, to ensure that the units accept emergency admissions.

a) Psychotic conditions characterized by severe hyperactivity or violence causing considerable risk to the patient's or another's life or health.

b) Psychotic or other conditions characterized by severe anxiety or depression, with considerable risk of a suicide attempt, or harm to the self or others.

c) Delirium, where detoxification is not a main issue.

d) Mental conditions in children and adolescents whom carers cannot handle and who need urgent help from CAMHS.

The services are required to provide mental health care without unnecessary delay 24 hours a day and seven days a week for adolescents in the 13-17-years age group in a specified population area. Each unit has a catchment area containing 17,000-31,000 inhabitants aged 13-17 years and is the only acute in-patient psychiatric service available for adolescents in its catchment area. The four units were located in three of the five health regions in Norway at that time. Treatment programmes were individualized from a range of elements, such as ward milieu therapy, individual psychotherapy, family therapy, medication, and the provision of specialized schooling. All the units had associated schools.

In 2005, Norwegian CAMHS treated 43,400 children and adolescents (4.0% of the population aged 0-17 years), 3,004 of whom were treated as day or in-patients in units with a total of 331 in-patient beds. In the same year, the total cost of CAMHS was 2,015 million NOK (2005 value). By the end of 2005, 3,203 full-time-equivalent employees worked in the CAMHS, 1,635 (51%) of whom worked at day and in-patient units [41].

### Patients

This study had a naturalistic and prospective cohort design. We collected pre-post data from the first episode of care, which started in 2005 for all patients, at four of the 16 acute in-patient psychiatric services for adolescents in Norway. Any later episode of care for the same patient was not included in the study. Two of the units admitted a few patients from a waiting list as well as acute patients, but only patients admitted immediately or within seven days of referral were included in the study. We excluded five patients from the study sample because they had waited more than seven days. Only 13 patients in the sample were not admitted on the day of referral, of whom 10 waited until the day after referral, two waited for four days (no/low suicidal risk; one had bipolar disorder, and one had externalizing problems), and one waited for five days (eating disorder, no/low suicidal risk).

### Measures

We developed a scoring manual for the study. This included data from the ordinary patient records and additional data obtained by the clinicians. *Involvement with the Child Protection Service *was coded "Yes" or "No" at intake. Living arrangements for the adolescents were scored in six categories: *living with both parents (biological or adoptive)*; - *with one parent*; *- with one parent and one stepparent*; *- in an institution*; *- alone or with others*; or *- in foster care*. At intake, the clinicians assessed and coded any suicidal problems as *suicide attempts before intake*, *specific suicidal plans*, *suicidal ideation without any associated plans*, *passive wish to die*, or *no suicidal problems*. Diagnoses were recorded according to the ICD-10 Mental and Behavioural Disorders [42] as assigned by the local clinical team. The patients were grouped into one of five different groups, according to their main diagnosis from Axis One [43]: *psychotic disorders *(F20-F29), *affective disorders *(F30-F39), *neurotic and stress-related disorders *(F40-F48, F93, F94), *eating disorders *(F50), and *externalizing disorders *(substance use F10-F19, personality F60-F69, hyperkinetic F90, conduct F91-F92, and "tics" F95). A sixth group included patients with no Axis One diagnosis. We also registered whether or not the patient had a *developmental disorder *(F70-F79, F80-F89).

The clinicians who were responsible for the patients used the HoNOSCA [21] to measure the adolescents' types and severity of mental health symptoms and problems. The HoNOSCA consists of 15 scales, but we used only scales 1-13 in this study. We did not use scale 14 and 15 because the inter-rater reliability of these two scales had been debated [44] and are not included in the HoNOSCA *total score*. Scale 1-13 focus on clinically significant problems and symptoms, and each scale is rated from 0 (no problem) to 4 (severe to very severe problem). The HoNOSCA *total score *is the sum of the scores for these 13 scales (range 0-52), and indicates the severity of the mental health problems [26]. The recommended rating period is the previous two weeks. The HoNOSCA has been evaluated well and its application found to be feasible in routine clinical settings [22-28,44]. It has been widely used in CAMHS as a routine measure of outcome [28,34-38] and has been used in several in-patient studies [8,29-33]. The clinicians working in the units participated in a three-hour standard training session in the use of the HoNOSCA before data collection was commenced. We investigated the inter-rater reliability of the HoNOSCA two months after the end of the data collection period for 61 of the 83 clinicians working at the four units. For this, we followed the procedures described by Hanssen-Bauer et al. [24] and used the same 20 written vignettes, including the provision that each clinician rated 10 of the 20 vignettes (either vignette group 1 or 2). We found moderate (intra-class correlation coefficient, ICC = 0.76) reliability for the HoNOSCA *total score *(the ICC ranged from 0.71 to 0.81 at the four units). The reliability was fair to substantial (0.41 to 0.95) for the 13 individual scales, and was lowest for scale 6 *physical illness, disability problem *(ICC = 0.41); scale 9 *emotional symptoms *(ICC = 0.52); scale 12 *family problems *(ICC = 0.53), and scale 5 *scholastic or language skills problems *(ICC = 0.58).

To determine the duration of hospitalization, we measured each in-patient episode as the days from intake until discharge (including when the patient was discharged after the last inclusion date, 31 December 2005).

The clinicians rated HoNOSCA at two time points for each patient: the first at intake (within 72 hours of arrival, the scoring period being the previous two weeks), and the second on the day of discharge (the scoring period being the previous three days) or on 28 February 2006 if not discharged by that date (N = 1). The clinicians did not rate HoNOSCA at the second time point if the discharge occurred three or fewer days after intake.

### Statistical analysis

We used SPSS 15.0 for Windows http://www.spss.com/ for the statistical analyses. Descriptive and test statistics were computed according to measurement type and distribution. All significance tests were two-tailed, and the level of significance was set at 0.05. To analyse the inter-rater reliability of the HoNOSCA, we computed ICC from variance components using the general linear model (GLM), as described by Hanssen-Bauer et al. [24]. We used Shrout's standards for the reliability results: *virtually none*: 0.00-0.10; *slight*: 0.11-0.40; *fair*: 0.41-0.60; *moderate*: 0.61-0.80; and *substantial *0.81-1.0 [45].

We computed the *change in the *HoNOSCA *total score *as the *intake *HoNOSCA *total score *minus the *discharge *HoNOSCA *total score *for each patient. Positive change scores thus indicated improvement, whereas negative change scores indicated deterioration. We calculated the effect size of the repeated measure (pre-post) difference in two ways: 1) Cohen's *d*: as the mean of the change scores divided by the standard deviation of the change scores [46]; and 2) as partial η^2 ^from the repeated measures using the GLM. We presented the severity scores on the 13 HoNOSCA scales as percentages, with *moderate problem *(score 3) or *severe/very severe problem *(score 4). We studied the main effects of the predictors of *change in *HoNOSCA *total score *as the main effects in an analysis of variance (ANOVA) model using the GLM in SPSS. We included the variables *unit*, *age*, *sex*, *Child Protection Service involved at intake*, *living with both parents*, *Axis One diagnosis*, *developmental disorder*, *intake *HoNOSCA *total score*, and *length of stay *as predictors.

The Regional Ethical Committees approved the study and the Norwegian Data Inspectorate licensed the study.

## Results

Data were available for 192 patients (70% girls) admitted to the four units in 2005 (40-62 patients in each unit). The data for 28 patients were missing, giving a response rate of 87%. The mean age of the 192 patients at intake was 15.7 years (SD 1.4, range 10-18 years). Four patients were < 13 years and two were 18. Patient ages did not differ between the units (F = 0.891; d.f. = 3, 188; *P *= 0.447). For 6% of the patients, both parents were from outside Europe (in an additional 6% of patients, one parent was from outside Europe). Living arrangements (31% lived with both parents) and the proportion of patients that were involved with the Child Protection Service at intake (24%) did not differ between the units. Table 1 shows other patient characteristics.

**Table 1:** Characteristics of the 192 patients

		Test of differences between the four units		
	**Proportion**	**χ**^**2**^	**d.f.**	**P**^**b**^

Sex (female)	70%	3.6	3	0.306
Living arrangements:		28.8	21	0.120
With one parent	38%			
With both parents	31%			
With one parent and one stepparent	10%			
Institution	10%			
Alone or with others	6%			
Foster care	4%			
Child Protection Service involved at intake	24%	6.1	3	0.108
Suicidal problems at intake:		10.7	12	0.556
Suicide attempt before intake	10%			
Specific suicidal plans	10%			
Suicidal ideation without any associated plans	38%			
Passive wish to die	12%			
No suicidal problems	26%			
Missing	4%			
Influenced (probably/definitely) by substances at intake	10%	1.7	3	0.635
Main disorder^a ^(grouped):		22.5	15	0.095
No disorder from Axis One	16%			
Affective	28%			
Externalizing	26%			
Neurotic	18%			
Psychosis	11%			
Eating	2%			
Developmental disorder:	14%	1.7	3	0.629

^a ^Main disorder from Axis One in the ICD-10 Multiaxial Classification [43].

^b ^We did not correct the *P *values for multiple comparisons.

### Mental health symptoms and problems at intake

The staff noted suicidal problems at intake in 113 (58%) of the 192 patients (suicide attempt before intake, specific suicide plans, or suicidal ideation without any associated plans). The unit staff judged that 10% of the patients were under the influence of substances at intake. Suicide problems and the influence of substances at intake did not differ between the units (Table 1). The units used standardized diagnostic interviews for 20% of the patients, but this practice differed (from 11% to 38%) between the units (χ^2 ^= 13.4; d.f. = 3; *P *= 0.004). Table 1 shows the distributions of the main mental or behavioural disorders (Axis One) and the frequency of *developmental disorders *(which did not differ between units). Table 2 shows the mental health problems at intake as the mean HoNOSCA *total scores*, and as the percentage of patients with score 3 (moderate) or score 4 (severe/very severe problems) on the different HoNOSCA scales. The mean HoNOSCA *total scores *did not differ between the units. On a test of the differences between units, one of the 13 HoNOSCA scales (HoNOSCA scale 2 *overactive, attention difficulties*) had a *P *value below 1% (without correction for multiple comparisons). The HoNOSCA scale most frequently rated as 3 or 4 was scale 9 *emotional symptoms *(81%). Table 3 shows the HoNOSCA *total scores *in the subgroups at intake. The total score was higher in boys than in girls, in those with compulsory (versus voluntary) admission, and in those with developmental disorders. It differed significantly across the diagnostic groups, being highest in the psychosis group. The HoNOSCA *total score *did not correlate significantly with age (*r *= -0.095; *P *= 0.189).

**Table 2:** Mental health problems at intake.

		Test of differences between the four units	
**HoNOSCA scales:**	**Score 3 or 4**	**χ^2 ^(3)^a^**	***P* ^c^**

1. Disruptive, aggressive problems	32%	0.4	0.939
2. Overactive, attention difficulties	31%	11.8	0.008**
3. Self-injury	38%	2.0	0.581
4. Alcohol, drug misuse	13%	7.7	0.052
5. Scholastic or language skills problems	21%	5.6	0.130
6. Physical illness, disability problems	8%	6.5	0.090
7. Hallucinations, delusions	24%	3.6	0.307
8. Psychosomatic problems	8%	7.1	0.067
9. Emotional symptoms	81%	1.0	0.806
10. Peer relationship problems	41%	7.9	0.048*
11. Self-care, independence problems	16%	3.4	0.332
12. Family problems	48%	2.4	0.490
13. Poor school attendance	38%	3.1	0.371

	Mean (SD)	F (3)^b^	
		
HoNOSCA *total score *(sum scale 1-13)	18.5 (6.4)	1.5	0.207

Percentages of patients (N = 192) with score 3 (moderate problem) or score 4 (severe/very severe problem) on each HoNOSCA scale and mean (SD) HoNOSCA total score.

^a ^Pearson χ^2 ^test of differences across the four services with the HoNOSCA five-scale scores reduced to dichotomous scale scores (0-2 and 3-4).

^b ^We tested differences in the HoNOSCA *total score *across the four services statistically with ANOVA.

^c ^We did not correct the *P *values for multiple comparisons.

* *P *< 0.05; ** *P *< 0.01.

**Table 3:** HoNOSCA total scores at intake according to subgroups

		HoNOSCA *total score *at intake		Test of differences between the subgroups		
	**N**	**Mean**	**SD**	***t***	**d.f.**	***P^c^***

All	192	18.5	6.4			
Sex:						
Female	135	17.7	6.5	2.4	190	0.015*
Male	57	20.2	5.9			
Living with both parents:						
Yes	60	18.9	6.7	0.6	190	0.521
No	132	18.3	6.3			
Child Protection Service involved at intake:						
Yes	46	19.1	6.2	0.7	190	0.475
No	146	18.3	6.5			
Admission:^a^						
Compulsory admission	41	21.0	6.1	3.5	121	0.001**
Voluntary admission	82	16.8	6.3			
Developmental disorder:						
Yes	27	22.7	6.2	3.9	190	< 0.001**
No	165	17.8	6.2			
Main disorder^b ^(grouped):				F	d.f.	*P*
				
No disorder from Axis One	31	17.3	7.0	4.6	5	0.001**
Psychosis	22	23.1	6.0			
Affective	53	16.9	5.5			
Neurotic	34	17.8	6.5			
Eating	3	12.0	2.0			
Externalizing	49	19.7	6.1			

^a ^Compulsory versus voluntary admission was only relevant and tested for age group ≥ 16 years (N = 123).

^b ^Main disorder from Axis One in the ICD-10 Multiaxial Classification [43]. We tested differences between these subgroups statistically with ANOVA.

^c ^We did not correct the *P *values for multiple comparisons.

* *P *< 0.05; ** *P *< 0.01.

### Hospitalization

According to the Norwegian Mental Health Act, patients who are 16 years or older can be subjected to compulsory admission to a mental health service. Of the 123 adolescents who were ≥ 16 years, 82 (67%) of the admissions were voluntary and 41 (33%) were compulsory. The compulsory admissions, which were based on a final decision made by the unit's consultant, differed significantly (range 7%-67%) between the units (χ^2 ^= 32.2; d.f. = 3; *P *< 0.001).

The median length of stay for the whole sample was 8.5 days (range 1-351 days), 25th percentile = three days, 75th percentile = 29 days. The length of stay was not associated with age (correlation Kendall's tau-b = -0.038, *P *= 0.491) or sex (Mann-Whitney *U *test = 3661.5, Z = -0.530, *P *= 0.596). The lengths of stay differed between the units (Kruskal-Wallis test χ^2 ^= 17.3; d.f. = 3; *P *= 0.001), with medians ranging from 4.5 days to 17 days. The clinic with the lowest median stay (4.5 days) also had the lowest maximum length of stay (51 days). The diagnostic groups also differed in the length of stay (Kruskal-Wallis test χ^2 ^= 30.2; d.f. = 5; *P *< 0.001). *Psychosis *had highest median stay of 37 days and *no diagnosis *had the lowest median stay of three days.

### Outcome of mental health symptoms and problems during admission

None of the patients died during hospital admission. In total, 136 patients had valid scores for mental health (HoNOSCA) at both time points (the second time point was more than three days after the first). The other 56 patients in the sample did not have HoNOSCA scores at discharge because they were hospitalized 3 days or less which was too short to measure outcome. For the 136 patients with outcome data, the mean HoNOSCA *total score *was 18.7 (SD 6.3) at the first time point and 13.6 (SD 7.1) at the second time point (paired sample *t *= 9.53; d.f. = 135; *P *< 0.001). The effect size was large (Cohen's *d *= 0.81 and partial η^2 ^= 0.40; *P *< 0.001). The effect sizes for the units differed considerably (Cohen's *d *from 0.49 to 1.40 and partial η^2 ^for the interaction *outcome *× *unit *was 0.11; *P *< 0.001). Table 4 shows the outcomes as percentages of patients with score 3 (moderate) or score 4 (severe/very severe problems) on the different HoNOSCA scales and the mean HoNOSCA *total score*. The HoNOSCA scales with the largest reduction in percentage points (for score 3 or 4) from intake to discharge were scale 3 *self-injury *(reduced from 38% of the adolescents to 9%) and scale 9 *emotional symptoms *(reduced from 82% of the adolescents to 54%). However, a large proportion of patients still had a score of 3 or 4 on the *emotional symptoms *scale at discharge. The HoNOSCA scale that showed the least change was scale 5 *scholastic or language skills problems *(no change).

**Table 4:** Mental health outcomes.

	Percentage with score 3 or 4		Test of differences (intake-discharge)^b^
**HoNOSCA scales**	**At intake**	**At discharge^a^**	***P* ^c^**

1. Disruptive, aggressive problem	24%	7%	< 0.001**
2. Overactive, attention difficulty	33%	18%	0.001**
3. Self-injury	38%	9%	< 0.001**
4. Alcohol, drug misuse	14%	2%	< 0.001**
5. Scholastic or language skills problem	19%	19%	1.000
6. Physical illness, disability	7%	4%	0.180
7. Hallucinations, delusions	29%	16%	0.001**
8. Psychosomatic problem	10%	3%	0.012*
9. Emotional symptoms	82%	54%	< 0.001**
10. Peer relationship problem	44%	29%	< 0.001**
11. Self-care, independence problem	16%	13%	0.388
12. Family problem	48%	37%	0.021*
13. Poor school attendance	37%	13%	< 0.001**

	Mean (SD)		

HoNOSCA *total score *(sum scale 1-13)	18.7 (6.3)	13.6 (7.1)	< 0.001**

Outcomes, as the change in mental health from intake to discharge, of the patients (N = 136)  who were admitted for more than three days. The percentages of patients with score 3  (moderate problem) or score 4 (severe/very severe problem) on each HoNOSCA scale and the  mean (SD) HoNOSCA *total scores*.

^a ^Median number of days between scores was 15 days (minimum four days and maximum 351 days).

^b ^Scale 1-13: based on the McNemar two related samples test. HoNOSCA total score: based on paired t tests: t = 9.5; d.f. = 135.

^c ^We did not correct the P values for multiple comparisons.

* P < 0.05; ** P < 0.01.

### Predictors of change during admission

We used univariate ANOVA models (in GLM) to analyse the predictors of *change in *HoNOSCA *total score*. We tested the variables in Table 3 (*sex*, *living with both parents*, *Child Protection Service involved at intake*, *Axis One diagnosis*, and *developmental disorder*), but not *compulsory or voluntary admission *because compulsory admission was only relevant to the age group ≥ 16 years. *Unit, age, intake *HoNOSCA *total score*, and *length of stay *were also included as predictors. We did not consider collinearity between the independent variables to be a problem because all the bivariate correlations were between -0.4 and 0.4. *Unit*, *intake *HoNOSCA *total score*, and *Child Protection Service involved at intake *were the only significant predictors. The findings are presented in Table 5. All predictors are shown, including those without significant effects. Altogether, the independent variables in the resulting model explained 25% of the variance (adjusted R^2^) in *change in *HoNOSCA *total score*. If only the significant predictors were included, their B values were similar to those of the full model, and the adjusted R^2 ^was 0.24.

**Table 5:** Predictors of change during admission.

Independent variable^b^	F	**d.f**.	*P*	B^c^	SE
Unit	7.5	3	< 0.001**		
Sex	< 0.1	1	0.898		
Age	1.1	1	0.296		
Child Protection Service involved at intake	4.8	1	0.031*	2.78^d^	1.27
Living with both parents	0.3	1	0.592		
Axis One diagnosis	1.3	5	0.282		
Developmental disorder	0.1	1	0.822		
Intake HoNOSCA total score	19.7	1	< 0.001**	0.37	0.08
Length of stay	0.8	1	0.363		

Univariate analysis of variance (general linear model) with change in HoNOSCA total score^a ^as the dependent variable.

^a ^*Change in *HoNOSCA *total score *> 0 represents improvement.

^b ^Model: R^2 ^= 0.34 and Adj R^2 ^= 0.25.

^c ^We present B values (unstandardized) for significant variables only.

^d ^For this variable: "No" was coded 0, "Yes" was coded 1.

* *P *< 0.05; ** *P *< 0.01.

## Discussion

In this study, we investigated patients admitted to four acute in-patient psychiatric units for adolescents in Norway. All the units accepted around-the-clock emergency admissions of adolescents living in a defined geographical area. The adolescent or caregiver could not choose another provider, so a possible self-selection of unit by the patients or caregivers was negligible in this system of care. We found that the four units treated patients who were comparable at intake. The high proportion of girls (70%) probably reflects the fact that the adolescents were often admitted with problems of self-harm and depression, and these problems are more common in female adolescents [47]. The proportion of compulsory admissions varied greatly between units. This has also been found in other countries, and more knowledge about such variations is needed [48,49]. Studies of adult psychiatric hospitals in Norway have found substantial regional differences in compulsory admissions, mainly explained by variations in diagnosis [50]. The length of stay was short in all the units. Differences in the length of stay between the units may reflect differences between the hospitals' resources for outpatient CAMHS and other local mental health services.

### Mental health at intake

The high frequency of suicidal problems, emotional symptoms, and affective and externalizing disorders at intake in all the units in our study was as expected for acute in-patient psychiatric services for adolescents. Our finding that the units used standardized diagnostic interviews for different proportions (11%-38%) of their patients may reflect differences in diagnostic competence or in the implementation of systematic assessments at the acute units. This may have resulted in reduced reliability of the diagnoses. However, the main diagnoses, type or severity of mental health symptoms, and problems at intake (HoNOSCA) did not differ between the units.

The adolescents in our sample had severe mental health problems, with a mean HoNOSCA *total score *of 18.5 at intake. This is similar to the findings of other studies of adolescents in in-patient services assessed with the HoNOSCA *total score *at admission [8,12,13,29-31,33] and higher than found in outpatient samples with mean HoNOSCA *total score *of 10.8-13.1 at intake [22,26,27]. We found that boys, those with compulsory admissions, and those with developmental disorders had significantly higher mean HoNOSCA *total scores *at intake, and that the HoNOSCA *total scores *differed according to the main Axis One diagnosis (highest if the main disorder was a psychotic disorder). These results were as expected and similar to those of an Australian study of adolescents under medium- to long-term in-patient mental health treatment [30]. In agreement with our findings, that study found no association between age and the HoNOSCA *total score *at intake and that patients with psychotic disorders had higher mean scores than those of patients with other disorders.

### Outcome at discharge

Overall, the magnitude of change in the HoNOSCA *total score *was large, which indicates that the patients' conditions were stabilized. However, this measures the pre-post change during admission without any comparison group.

The HoNOSCA *total score *at intake was a strong positive predictor of outcome (improvement), as expected and as reported by others [23,32], but the lack of a control group should be kept in mind when interpreting our results [51]. Length of stay was not related to outcome. The possible mechanism underlying this observation may be that the units admitted patients when they were in an acute crisis and successfully adjusted the length of stay to ensure that outpatient services or primary care was appropriate for the patients. This interpretation is supported by our finding that the severity of the mental health problem at discharge, as measured by the mean HoNOSCA *total score*, was 13.7, which is close to the scores reported by others for outpatient groups [26,27].

A British study [8] of 55 children aged 6-17 years, who were hospitalized for a longer period (median stay 21.6 weeks) than the patients in our sample, found that process variables, such as therapeutic alliance and family functioning, predicted health gain, rather than the presenting symptoms. Two other British in-patient studies [12,13] also found that outcomes were unrelated to diagnoses. One of these studies [12] reported that neither the length of stay nor sex predicted outcome, which is consistent with our results. They also found that the involvement of a child protection service was not significantly associated with outcome, in contrast to our findings. We cannot explain why the involvement of the Child Protection Services at intake was a positive, independent predictor of change in the multivariate analysis. Involvement of CPS indicates that a family needs support and our finding seems to be contrary to the findings by others that better premorbid family functioning predicts good outcome [4,5,8,10]. Our finding is puzzling, and the significance of the adolescent's system of care deserves further attention.

Our findings that the HoNOSCA scales that showed the greatest reductions were scale 3 *self-injury *and scale 9 *emotional problems *and that the HoNOSCA scale with the least change was scale 5 *scholastic or language skills problems *(no change) were as expected for these acute in-patient psychiatric services. We also found a large reduction in poor school attendance, which may be a result of the hospitals providing specialised education. The rating instructions for the HoNOSCA [21] state that attendance at hospital school should be included as school attendance when rating HoNOSCA scale 13 *poor school attendance*. A reduction in poor school attendance during hospital stay may therefore artificially indicate improvement of mental health. However, we found similar results when analysing effect sizes (outcome) and predictors of change during admission without including the HoNOSCA scale 13 *poor school attendance *in the HoNOSCA *total score*.

The finding that patients at the different units achieved significantly different outcomes warrants further evaluation of the service and treatment quality. The effect sizes differed considerably between the units. Systematic rater bias could have contributed to this finding. However, we found moderate inter-rater reliability for the HoNOSCA *total score *among the clinicians that participated in this study, in agreement with another study of the inter-rater reliability of HoNOSCA in outpatient clinics [24] and with a cross-national study [25]. Lyons and McCulloch also found differences in outcome between child and adolescent in-patient units, and they recommended monitoring and comparing the outcomes of in-patient units to identify their strengths and weaknesses.

### Strengths and limitations

The lack of a comparison group and the possibility of informant bias when the staff themselves rated the HoNOSCA for their own patients are limitations of this study. However, from an audit perspective, the easy collection of information as part of ordinary clinical practice was essential, and there was a trade-off between the feasibility of the study and a more elaborate design. It is less probable that "drifting" (increased systematic error with time) in the ratings influenced our outcome results because the intakes occurred throughout a whole year and the median length of stay was short. The in-patient units investigated in this study had not described their treatment programmes thoroughly, and these probably differed in some respects. This may limit the generalizability of the results. We also lack data on the adolescents' history of mental disorders and previous treatment. The strengths of this study were the low probability that the patients self-selected the units (also reflected in the similarities in the patient characteristics at intake between the units); the high number of participants; the high response rate; and the use of an established clinician-rated mental health measure, the HoNOSCA, with satisfactory inter-rater reliability among the clinicians who participated in the study.

### Future research

There is a lack of knowledge about in-patient acute psychiatric services for adolescents while in many countries providers establish such services. There is a need to know more about the therapeutic content and the outcomes of these services, compared to alternatives to admission.

## Conclusions

The acute adolescent psychiatric in-patient services admitted young people with severe mental health problems, the majority of whom had suicidal problems and 70% of whom were girls. Although the median stay was short (8.5 days), we found a large improvement in their mental health problems, to levels appropriate for their return to the community and further outpatient care if required. This is in accordance with the aims of these services. We found that the different units admitted similar adolescents for in-patient treatment, but showed significant differences between the units in the proportion of compulsory admissions, lengths of stay, and treatment outcomes. This indicates differences in service delivery, so there may be potential for quality improvement if the reasons for these differences are explored further.

## Competing interests

The authors declare that they have no competing interests.

## Authors' contributions

KHB and SH analysed the results and drafted the paper. The other authors initiated the study and commented on the drafts. TH coordinated the sites. GJ, PMO, TS, and TT collected the data. All the authors have read and approved the final manuscript.
